# Effect of Cumulative Spirulina Intake on Broiler Meat Quality, Nutritional and Health-Related Attributes

**DOI:** 10.3390/foods13050799

**Published:** 2024-03-05

**Authors:** Maria P. Spínola, Mónica M. Costa, José A. M. Prates

**Affiliations:** 1 CIISA—Centro de Investigação Interdisciplinar em Sanidade Animal, Faculdade de Medicina Veterinária, Universidade de Lisboa, Av. da Universidade Técnica, 1300-477 Lisboa, Portugal; mariaspinola@fmv.ulisboa.pt (M.P.S.); monicacosta@fmv.ulisboa.pt (M.M.C.); 2 Associate Laboratory for Animal and Veterinary Sciences (AL4AnimalS), Av. da Universidade Técnica, 1300-477 Lisboa, Portugal

**Keywords:** *Arthrospira platensis*, broiler meat, cumulative intake, meat quality, microalgae, poultry

## Abstract

This work aimed to assess how different cumulative levels of Spirulina (*Arthrospira platensis*) intake influence individual broiler meat quality parameters, nutritional value and health-related traits. The data analysed showed varying cumulative Spirulina intake levels, ranging from 3.46 to 521 g/bird, with large changes in meat traits. The key findings indicate that Spirulina intake significantly enhances meat colour, primarily due to its rich carotenoid content. However, this enhancement shows a saturation effect at higher intake levels, where additional Spirulina does not further improve the colour. Regarding the meat nutritional profile, Spirulina increases beneficial *n* − 3 polyunsaturated fatty acids and reduces lipid oxidation. These effects on meat, however, are not linear and become more complex at higher microalga intake levels. Regarding meat sensory attributes, moderate Spirulina levels positively influence flavour and texture. Still, higher levels may lead to changes not universally preferred by meat consumers, highlighting the need for balanced Spirulina inclusion in diets. Optimal Spirulina cumulative intake levels must be identified to balance meat’s nutritional benefits with consumer preferences. Additionally, ensuring Spirulina’s purity and adherence to regulatory standards is essential for consumer safety and market access. These findings provide valuable insights for poultry nutritionists and the food industry, emphasising the necessity of a balanced approach to Spirulina’s incorporation in poultry diets.

## 1. Introduction

The quest for sustainable and health-promoting livestock feeds has intensified in recent years, emphasising natural additives and feedstocks that enhance the nutritional value and quality of animal products. *Arthrospira platensis*, known as Spirulina, belongs to the *Arthrospira* genus, which has other species (*Arthrospira maxima* [1] and *Arthrospira fusiformis* [2]). It is a microalga rich in proteins, vitamins and antioxidants and has emerged as a promising feed supplement and ingredient in poultry diets. Its inclusion in broiler diets has been studied extensively for its potential to improve meat quality, specifically in terms of nutritional value, sensory attributes and health-related compounds [3,4].

Spirulina stands out due to its high protein content (up to 76% of dry matter, DM) [5,6], essential amino acids, carbohydrates (up to 22.6% DM) [5] and unique pigment composition, which includes phycocyanin, carotenoids and chlorophylls [7,8,9]. These components not only enhance the growth performance of broilers but also positively impact meat quality [5]. Studies have shown that Spirulina inclusion can modify meat colour, an attribute largely influenced by its carotenoid content, leading to enhanced consumer appeal [10]. Moreover, the impact of Spirulina on the fatty acid profile and antioxidant capacity of broiler meat has garnered significant interest. The incorporation of Spirulina in broiler diets has been linked to increased levels of beneficial fatty acids, particularly *n* − 3 polyunsaturated fatty acids (PUFAs), and a reduction in lipid oxidation, as measured by lower thiobarbituric acid-reactive substance (TBARS) values [3]. This shift towards a healthier fatty acid composition and improved oxidative stability is crucial in the context of human nutrition and the shelf-life extension of meat products.

However, incorporating Spirulina into broiler diets presents a complex landscape of effects influenced by the level of its inclusion and the duration of feeding. This complexity is pivotal in determining the overall impact on meat quality. While moderate levels of Spirulina have been linked to beneficial changes in meat quality, higher levels may induce alterations in flavour or texture, which might not align with universal consumer preferences. High levels of Spirulina can also negatively affect growth performance, decreasing body weight gain and increasing the feed conversion ratio [9]. This highlights the critical need to identify the optimal levels of Spirulina inclusion and appropriate feeding durations that can harmonise the dual goals of nutritional enhancement and consumer acceptability. To achieve the optimal levels of Spirulina inclusion, further in vivo studies, particularly those comparing different microalga levels with various feeding durations, are necessary, in collaboration with industrial companies of poultry production. Moreover, the safety aspects and regulatory compliance associated with using Spirulina as a feed additive cannot be overstated. Ensuring that the Spirulina utilised in poultry diets is free of contaminants is essential for safeguarding animal and, consequently, human health. Also, adherence to established regulatory guidelines is fundamental in upholding consumer trust and securing market access [11].

In line with this, the primary aim of this research was to comprehensively investigate the effects of cumulative Spirulina intake on broiler meat traits. The cumulative intake was calculated as the total feed consumed by the bird throughout the experimental period multiplied by the proportion of microalgae in the diet. We hypothesised that these effects are a consequence of the specific transfer of compounds from the microalga to the meat, each following its distinct kinetics. More specifically, our research focused on evaluating how different levels of cumulative Spirulina intake impact various aspects of broiler meat quality (colour, pH 24 h and sensory attributes), nutritional value (total protein, total fat and selected fatty acids) and health-related traits (oxidation, carotenoids and cholesterol). The species considered for the present review was only *Arthrospira platensis* because, to the best of our knowledge, there are no reports available about the influence of other Spirulina species on the quality and nutritional traits or health-related compounds of broiler meat. However, several reports described the nutritional properties of *Arthrospira maxima* [12,13,14,15] and *Arthrospira fusiformis* [16,17], which indicates the potential use of these microalgae in poultry feed. Indeed, *A. maxima* is rich in high-quality protein, with values ranging from 44.9 [12] to 64.0% [14] dry weight, and in pigments, such as phycocyanin (14%) [13]. The nutritional composition of *A. maxima* could be further optimised by irradiation using light sources, leading to a protein content of up to 73.5%, which shows the response of this microalga to varying culture conditions [12]. In addition, *A. fusiformis*, depending on growth conditions, showed an interesting nutritional composition, with a protein content between 43.0 and 65.0% dry weight [16,17] and an amino acid profile with significant amounts of limiting amino acids for poultry feed, such as lysine (5.23%) and methionine (2.85%) [16].

The outcomes of this study are discussed in the broader context of safety, regulatory compliance and consumer acceptability, providing valuable insights for both poultry nutritionists and food industry stakeholders.

## 2. Impact of Cumulative Spirulina Intake on Broiler Meat Quality Traits

Table 1 provides a comprehensive overview of how cumulative Spirulina intake impacts broiler meat quality, focusing on the colour traits, ultimate pH (pH 24 h) values and sensory attributes. The data show varying cumulative Spirulina intake levels, ranging from 3.46 to 521 g/bird, with corresponding changes in meat traits.

Concerning meat colour traits, the initial increases in Spirulina intake from low levels like 3.46 g/bird led to noticeable enhancements in meat colour. However, at higher intake levels (221 and 230 g/bird), the rate of change in colour traits reached a plateau, suggesting a saturation point. This means that above these levels of Spirulina, the difference in Spirulina intake does not make a difference in colour traits. This observed saturation effect in colour traits aligns well with the findings of El-Bahr et al. [4] and Park et al. [8], indicating Spirulina’s potential to enhance meat colour through its natural pigments, especially carotenoids. This effect seems, in fact, to reach a saturation point, beyond which additional Spirulina intake does not further improve the colour, as also suggested by Toyomizu et al. [18].

Variations in pH 24 h did not follow a consistent pattern across Spirulina intake levels, indicating that factors other than Spirulina might significantly influence the meat’s ultimate pH. This inconsistent impact of Spirulina on meat pH agrees with the findings of Moustafa et al. [19] and indicates the complexity of factors influencing meat pH, with Spirulina intake being a less dominant factor. Other factors that influence pH are genetics, pre-slaughter stress, handling and transportation, antemortem exercise, slaughter age and nutrition, muscle type and carcass electrical stimulation [20].

Sensory qualities such as flavour and juiciness showed more pronounced changes at higher Spirulina intakes, suggesting a linear relationship without a clear saturation point. The linear relationship in sensory traits, with increasing Spirulina intake enhancing these qualities, is supported by the results of Pestana et al. [9]. However, unlike the colour traits, there is no evident saturation point, indicating continuous changes in sensory attributes with higher Spirulina intake levels.

In summary, a multifaceted impact of cumulative Spirulina intake on broiler meat quality is observed. While colour enhancements exhibit saturation kinetics, the effects on pH and sensory traits are more complex. The findings underscore the importance of determining optimal Spirulina intake levels in poultry diets, balancing the benefits of enhancing meat quality against the variability of impacts on different traits.

**Table 1:** Impact of cumulative Spirulina intake on quality traits of broiler meat.

Initial Age and Weight	Alga Level (% Feed) and Duration of the Trial ^3^	Cumulative Alga Intake (g/Bird) ^4^	Colour Traits						pH 24 h		Sensory Traits ^7^								References
			Absolute Value (CIELAB Scale) ^5^			Difference ^6^ (Alga–Control)			Absolute Value (pH Scale)	Difference ^6^ (Alga–Control)	Absolute Value (8 Points Scale)				Difference ^6^ (Alga–Control)				
			L*	a*	b*	L *	a*	b*			F	OF	J	T	F	OF	J	T	
4 d old, 74.1 g	0.10%, 32 d	3.46	-	-	-	-	-	-	5.93	0.19	-	-	-	-	-	-	-	-	[4]
1 d old, 41.5 g	0.25%, 34 d	7.22	45.5	10.1	9.64	3.89	−0.37	0.31	5.89	0.15	-	-	-	-	-	-	-	-	[8]
22 d old, 640 g	0.5%, 20 d	13.9	47.4	5.32	10.7	−1.10	0.8	6.3	5.80	0.04	-	-	-	-	-	-	-	-	[19]
1 d old, 41.5 g	0.5%, 34 d	14.4	45.1	10.8	9.93	3.54	0.36	0.60	5.85	0.11	-	-	-	-	-	-	-	-	[8]
1 d old, 41.5 g	0.75%, 34 d	21.8	43.2	11.1	8.46	1.58	0.63	−0.87	6.15	0.41	-	-	-	-	-	-	-	-	[8]
1 d old, 41.5 g	1.0%, 34 d	28.9	46.5	10.3	9.86	4.87	−0.15	0.53	6.15	0.41	-	-	-	-	-	-	-	-	[8]
22 d old, 640 g	1.0%, 20 d	28.9	46.8	5.48	11.6	−1.70	1.0	7.2	5.79	0.03	-	-	-	-	-	-	-	-	[19]
21 d old	4.0%, 16 days	29.8	45.4	3.4	6.30	−2.6	2.40	2.8	-	-	-	-	-	-	-	-	-	-	[18]
22 d old, 640 g	1.5%, 20 d	40.4	47.1	5.36	12.2	−1.40	0.9	7.8	5.74	−0.02	-	-	-	-	-	-	-	-	[19]
21 d old	8.0%, 16 days	59.5	48.1	1.6	12.3	0.10	0.60	8.8	-	-	-	-	-	-	-	-	-	-	[18]
21 d old	15%, 14 d	84.7	46.6	5.31	10.7	−2.60	0.78	6.32	5.77	−0.02	4.40	0.48	4.00	5.24	−0.12	0.17	0.50	0.14	[9]
7 d old, 132 g	15%, 28 days	221	57.7	4.99	20.9	−2.10	0.59	11.7	5.57	−0.24	5.48	0.20	4.56	5.00	−0.53	0.10	0.89	1.04	[21]
14 d old, 132 g	15%, 21 days	230	59.5	4.40	17.4	−2.60	0.21	9.81	5.48	−0.18	-	-	-	-	-	-	-	-	[22]
15 d old ^1^	10%, 20 d	254	53.9	5.77	23.7	−1.15	3.3	13.1	-	-	-	-	-	-	-	-	-	-	[23]
15 d old ^2^	10%, 22 d	315	54.8	6.13	23.9	0.15	3.20	13.3	-	-	-	-	-	-	-	-	-	-	[23]
1 d old	10.8%, 34 d	324	53.0	3.48	12.3	−2.39	1.05	0.98	6.06	0.26	-	15.8	-	24.4	-	−1.30	-	22.7	[11]
1 d old	17.3%, 34 d	521	57.4	3.81	15.1	0.40	2.02	2.00	5.99	0.03	59.1	11.1	-	-	3.10	−3.80	-	-	[24]

^1^ Female broilers. ^2^ Male broilers. ^3^ For the duration of the trial, the last day, corresponding to slaughtering, was not considered. ^4^ Calculated as the total feed ingested per animal during the experimental period multiplied by the proportion of microalgae in the diet. For some of the studies, no information about the cumulative feed intake (CFI) was available, and therefore, it was estimated as follows: CFI (g/bird) (Altmann et al. [11]; Altmann et al. [24]) = (CFI (Abdel-Moneim et al. [25]) + CFI (Ibrahim et al. [10]) + CFI (Neumann et al. [26]) + CFI (Park et al. [8]) + CFI (Sugiharto et al. [27]))/5; the studies had the same duration of trial and initial animal age; CFI (g/bird) (Moustafa et al. [19]) = CFI (g/d/bird) × number of days; CFI (g/bird) (Mullenix et al. [23]) = CFI (lb/bird) × 453.59237; CFI (g/bird) (Costa et al. [22]; Pestana et al. [9]; Spínola et al. [21]) = (CFI (g/d/pen) × number of days)/number of birds; CFI (g/bird) (Toyomizu et al. [18]) = CFI (Pestana et al. [9]) + 2× (CFI (Pestana et al. [9])/14). ^5^ L*: lightness; a*: redness; b*: yellowness. ^6^ The absolute values were used to calculate the difference between microalga and control results. ^7^ F, flavour; OF, off-flavour; J, juiciness; T, tenderness.

## 3. Influence of Cumulative Spirulina Intake on Broiler Meat Nutritional Attributes

Table 2 presents an insightful analysis of how cumulative Spirulina intake impacts the nutritional value of broiler meat, focusing on protein, fat and specific fatty acids. The data show varying cumulative Spirulina intake levels, ranging from 3.46 to 521 g/bird, with associated changes in meat traits.

Regarding the contents of protein and fat, the data vary across different Spirulina intake levels. For instance, at a 2.0% feed level (65.4 g/bird), Ibrahim et al. [10] reported an increase in protein content. However, other studies, like those by Bonos et al. [3] at 0.50% and 1.0% feed levels (21.5 and 41.3 g/bird), did not show significant changes in protein and fat contents. This variability suggests a complex relationship between Spirulina intake and these macronutrients. The relationship between Spirulina intake and changes in protein and fat contents is not linear. While some studies showed enhanced protein content at higher intake levels, others did not find significant changes. This aligns with findings from Altmann et al. [11] and Altmann et al. [24], indicating that the impact of Spirulina on protein and fat levels may depend on factors like the specific strain of Spirulina, the bird’s age and the overall diet composition. Indeed, a study by Fries-Craft et al. [28] showed that the effect of a microalga strain incorporated in a maize- or wheat-based diet at 0.175% and fed to broilers for 42 days was dependent on the basal diet’s composition since an improvement in animal growth performance was only observed with the maize-based diet. In the reviewed literature, the basal diet composition is also variable. For instance, a maize- and soybean-based diet was used by El-Bahr et al. [4], Pestana et al. [9], Ibrahim et al. [10] and Mullinex et al. [21], whereas maize and barley and maize, soybean and wheat were the basal ingredients applied by Toyomizu et al. [18] and Altmann et al. [11], Park et al. [8] and Altmann et al. [24], respectively. Moreover, the differences observed in the nutritional composition of broiler meat, particularly in terms of lipids and protein, can be attributed to the bioaccessibility and digestibility of Spirulina nutrients and not just to the composition of basal diets fed to broilers, which were similar between studies [10,11,24]. The bioaccessibility of Spirulina nutrients can be enhanced through the application of mechanical or enzymatic pre-treatments [29,30]. These pre-treatments allow the disruption of microalgal cell walls and the consequent release and hydrolysis of nutritional compounds [29,30]. A recent report suggested an increase in protein bioaccessibility from extruded Spirulina caused by a decrease in protein solubility, which was detected by a reduction in the amount of total protein released from algal biomass [29]. In addition, Costa et al. [30] observed an increase in the extraction and hydrolysis of the protein fraction with 18 to 26 kDa, probably corresponding to phycocyanin subunits, after pre-treating Spirulina with a combination of extrusion and pancreatin.

Spirulina is very rich in protein and well balanced in essential amino acids [17]. In a study performed by El-Bahr et al. [4], a supplementation of 0.10% microalga in broiler diets increased the levels of lysine, methionine, tryptophan, histidine and aspartic acid in the breast muscle.

The fatty acid profile, particularly *n* − 3 PUFAs like 18:3*n* − 3, 20:5*n* − 3 and 22:6*n* − 3, shows changes with varying levels of Spirulina intake. Bonos et al. [3] reported increases in certain *n* − 3 PUFAs at lower intake levels, while higher levels (221 and 230 g/bird) from studies by Spínola et al. [21] and Costa et al. [22] indicate more significant alterations in fatty acid profiles. This trend suggests Spirulina’s potential to enhance the nutritional quality of broiler meat through modifications in fatty acid composition. The consistent increase in certain *n* − 3 PUFAs at various Spirulina intake levels agrees with the literature findings [3,9]. This suggests that Spirulina can positively influence the fatty acid profile of broiler meat, enhancing its nutritional value. The changes in fatty acid composition at higher intake levels indicate that Spirulina might be particularly effective in modifying the fatty acid profile, though the specific relationship could be influenced by other dietary factors.

Spirulina can influence meat quality at a biochemical level in poultry as a source of antioxidants and essential fatty acids [31]. β-Carotene and tocopherol are relevant as antioxidants, since they can help neutralise oxidative stress in tissues and prevent lipid oxidation. A better antioxidant status could contribute to an improvement in meat quality by reducing the development of off-flavours and improving colour stability [31]. Fatty acids, such as γ- and α-linolenic acids, with an important role in membrane structure and function, can influence lipid composition and, therefore, meat texture and flavour [31]. 

Overall, the findings indicate that cumulative Spirulina intake can influence the nutritional value of broiler meat, especially in terms of fatty acid composition. This is significant from a health perspective, considering the health benefits of *n* − 3 PUFAs. In turn, the effects on protein and fat contents are less predictable and do not follow a straightforward pattern. This complexity highlights the need for further research to fully understand the nuances of how Spirulina intake affects broiler meat’s nutritional profile.

**Table 2:** Impact of cumulative Spirulina intake on nutritional value of broiler meat.

Initial Age and Weight	Alga Level (% Feed) and Duration of the Trial ^1^	Cumulative Alga Intake ^2^ (g/Bird)	Chemical Composition														References
			Absolute Value							Difference ^5^ (Alga–Control)							
			Protein (%) ^3^	Fat (%) ^4^	Fatty Acids (% Total Fatty Acids)					Protein (%)	Fat (%)	Fatty Acids (% Total Fatty Acids)					
					18:2*n* − 6	18:3*n* − 3	20:5*n* − 3	22:5*n* − 3	22:6*n* − 3			18:2*n* − 6	18:3*n* − 3	20:5*n* − 3	22:5*n* − 3	22:6*n* − 3	
4 d old, 74.1 g	0.10%, 32 d	3.46	-	-	21.3	1.98	2.77		3.03	-	-	−2.76	−0.27	1.57	-	1.57	[4]
1 d old	0.50%, 41 d	21.5	-	-	28.5	2.12	0.22	0.77	0.78	-	-	2.13	0.40	−0.02	−0.10	−0.08	[3]
1 d old	1.0%, 41 d	43.1	-	-	26.4	1.94	0.22	0.78	0.72	-	-	0.02	0.22	−0.01	−0.09	−0.14	[3]
1 d old	2.0%, 34 d	65.4	24.7	0.26	-	-	-	-	-	2.06	−0.10	-	-	-	-	-	[10]
21 d old	15%, 14 d	84.7	-	1.36	18.0	0.65	0.18	0.61	0.47	-	0.03	−2.60	−0.39	−0.02	−0.02	0.08	[9]
7 d old, 132 g	15%, 28 days	221	-	0.81	21.5	0.56	0.14	0.60	0.46	-	−0.54	−7.60	0.11	0.10	0.38	0.31	[21]
14 d old, 132 g	15%, 21 days	230	-	1.10	22.7	0.63	0.10	0.47	0.28	-	−0.49	−9.60	0.16	0.08	0.27	0.16	[22]
1 d old	10.8%, 34 d	324	21.6	3.12	-	-	-	-	-	1.08	−0.28	-	-	-	-	-	[11]
1 d old	17.3%, 34 d	521	21.6	2.70	37.4	3.06	0.12	-	0.57	0.09	0.00	−1.60	−0.37	−0.05	-	−0.15	[24]

^1^ For the duration of the trial, the last day, corresponding to slaughtering, was not considered. ^2^ Calculated as the total feed ingested per animal during the experimental period multiplied by the proportion of microalgae in the diet. For some of the studies, no information about the cumulative feed intake (CFI) was available, and therefore, it was estimated as follows: CFI (g/bird) (Altmann et al. [11]; Altmann et al. [24]) = (CFI (Abdel-Moneim et al. [25]) + CFI (Ibrahim et al. [10]) + CFI (Neumann et al. [26]) + CFI (Park et al. [8]) + CFI (Sugiharto et al. [27]))/5; the studies had the same duration of trial and animal initial age; CFI (g/bird) (Bonos et al. [3]) = (CFI (Abbas et al. [32]) + CFI (Abou-Zeid et al. [33]) + CFI (Feshanghchi et al. [34])/3; the studies had the same duration of trial and animal initial age; CFI (g/bird) (Costa et al. [22]; Pestana et al. [9]; Spínola et al. [21]) = (CFI (g/d/pen) × number of days)/number of birds. ^3^ Determined using a FOSS FoodScan Near-Infrared Spectrophotometer, as described by Anderson [35]. ^4^ Ibrahim et al. [10], Altmann et al. [11] and Altmann et al. [24] used a FOSS FoodScan Near-Infrared Spectrophotometer, as described by Anderson [35], whereas Pestana et al. [9], Spínola et al. [21] and Costa et al. [22] performed lipid extraction according to Folch et al. [36]. ^5^ The absolute values were used to calculate the difference between microalga and control results.

## 4. Effect of Cumulative Spirulina Intake on Broiler Meat Health-Related Compounds

Table 3 presents data on the impact of cumulative Spirulina intake on health-related compounds in broiler meat, specifically focusing on TBARS (an indicator of oxidative stress), total carotenoids and cholesterol levels. The data show varying cumulative Spirulina intake levels, ranging from 3.46 to 521 g/bird, with related alterations in meat traits.

TBARS are used to measure oxidative stress in meat, with lower values indicating better oxidative stability. The data show a decrease in TBARS values at lower Spirulina intakes (21.5 and 43.1 g/bird, as per Bonos et al. [3]), suggesting that Spirulina can help in reducing oxidative stress in meat. However, at higher intakes (e.g., 221 and 230 g/bird from unpublished studies by Spínola et al. [21] and Costa et al. [22]), the TBARS values were higher, indicating a potential threshold beyond which Spirulina might not effectively reduce oxidation. The reduction in TBARS at lower Spirulina intakes aligns with the antioxidative properties of Spirulina, as reported by Bonos et al. [3]. However, the increased TBARS values at higher intakes suggest a complex relationship between Spirulina and oxidative stability, possibly due to an imbalance in antioxidant mechanisms at higher intake levels.

An increase in total carotenoids was observed across various Spirulina intake levels. The increase in total carotenoids across various Spirulina intake levels indicates a more consistent, possibly linear relationship. Higher Spirulina intake consistently leads to higher carotenoid levels in the meat, reflecting the rich carotenoid content of Spirulina and its transfer to the meat. This is significant, as carotenoids are potent antioxidants, and their increased levels in meat can indicate improved nutritional value and oxidative stability. The consistent increase in carotenoids across different Spirulina intake levels agrees with the findings of Pestana et al. [9]. This suggests that Spirulina, being rich in natural antioxidants, can enhance the carotenoid content in broiler meat, thus potentially improving its nutritional quality.

The cholesterol levels in meat are a nutritional concern, and the data indicate that higher Spirulina intake can influence these levels. However, the direction and magnitude of this influence vary across different intake levels and studies. The varying impact of Spirulina on cholesterol levels, noted in the studies by Pestana et al. [9] and the unpublished studies [21,22], indicates that Spirulina’s influence on meat cholesterol is not straightforward. The varying results suggest that other factors, possibly including the bird’s diet composition and genetic factors, might play a role in how Spirulina intake affects cholesterol levels in meat.

Summing up, while there is a discernible relationship between cumulative Spirulina intake and health-related compounds in broiler meat, this relationship varies depending on the specific compound. For oxidative stability (TBARS) and cholesterol levels, the relationship is non-linear and influenced by other factors. In contrast, for total carotenoids, a more consistent linear relationship is observed, where increased Spirulina intake correlates with increased carotenoid levels in the meat. These insights highlight the complex interactions between Spirulina intake and broiler meat quality, underscoring the importance of optimising Spirulina intake to maximise health benefits while considering potential limitations and variable impacts on different health-related compounds.

**Table 3:** Impact of cumulative Spirulina intake on health-related compounds of broiler meat.

Initial Age and Weight	Alga Level (% Feed) and Duration of the Trial ^1^	Cumulative Alga Intake ^2^ (g/Bird)	Health- and Antioxidant-Related Compounds								References
			Absolute Value				Difference ^5^ (Alga–Control)				
			TBARS ^3^ (mg Malonaldehyde/kg)		Total Carotenoids (µg/g)	Cholesterol (mg/g) ^4^	TBARS ^3^ (mg Malonaldehyde/kg)		Total Carotenoids (µg/g)	Cholesterol (mg/g) ^4^	
1 d old	0.50%, 41 d	21.5	0.14 (d2)	0.43 (d5)	-	-	−0.008	0.15	-	-	[3]
1 d old	1.0%, 41 d	43.1	0.11 (d2)	0.33 (d5)	-	-	−0.035	0.05	-	-	[3]
1 d old	2.0%, 34 d	65.4	-	-	-	12.4	-	-	-	−3.65	[35]
21 d old	15%, 14 d	84.7	0.25 (d0) 0.27 (d2)	0.27 (d6)	161	0.67	−0.018 (d0) −0.12 (d2)	−0.13	112	0.051	[9]
7 d old, 132 g	15%, 28 days	221	0.18 (d0)	0.27 (d8)	242	0.61	0.010	−0.040	213	−0.18	[21]
14 d old, 132 g	15%, 21 days	230	0.31 (d0)	0.52 (d8)	172	0.50	0.00	0.28	145	−0.090	[22]
1 d old	10.8%, 34 d	324	0.095 (d0)	-	-	-	−0.003	-	-	-	[11]

^1^ For the duration of the trial, the last day, corresponding to slaughtering, was not considered. ^2^ CFI (g/bird) (Altmann et al. [11]) = (CFI (Abdel-Moneim et al. [25]) + CFI (Ibrahim et al. [10]) + CFI (Neumann et al. [26]) + CFI (Park et al. [8]) + CFI (Sugiharto et al. [27]))/5; the studies had the same duration of trial and animal initial age; CFI (g/bird) (Bonos et al. [3]) = (CFI (Abbas et al. [32]) + CFI (Abou-Zeid et al. [33]) + CFI (Feshanghchi et al. [34]))/3; the studies had the same duration of trial and animal initial age; CFI (g/bird) (Costa et al. [22]; Pestana et al. [9]; Spínola et al. [21]) = (CFI (g/d/pen) × number of days)/number of birds. ^3^ Thiobarbituric acid-reactive substances. ^4^ Ibrahim et al. [10] measured it at a wavelength of 578 nm using a spectrophotometer, whereas Costa et al. [22], Pestana et al. [9] and Spínola et al. [21] measured it at a wavelength of 202 nm using a high-performance liquid chromatography (HPLC) system with UV–visible photodiode array detection. ^5^ The absolute values were used to calculate the difference between microalga and control results.

## 5. Safety Precautions and Consumer Acceptability of Broiler Meat

In examining the safety precautions and consumer acceptability of broiler meat associated with Spirulina intake, several crucial aspects emerge. First, the safety of Spirulina as a feed additive or ingredient is generally acknowledged, particularly when it is free of contaminants. Spirulina is generally considered safe by the European Food Safety Authority, and, according to Martin-Girela et al. [37], several contaminants from freshwater were present in Spirulina below the detection limit, which makes Spirulina safe for consumption. Moreover, Grosshagauer et al. [38] reported that mercury, aluminium, cadmium and arsenic were below the maximum allowed intake levels in previously analysed Spirulina samples, and, although lead was found at levels above the maximum limit of 3 mg/kg, these results occurred in less than half of the analysed samples. As highlighted in studies like El-Bahr et al. [4], ensuring that Spirulina is devoid of heavy metals or harmful microorganisms is essential, as these can pose significant health risks. While Spirulina is well regarded for its safety when pure and well processed, rigorous quality checks are critical. Indeed, it is possible to detect microcystins in Spirulina used as feed supplements for fish [39], but with the proper cultivation and production conditions assuring a detection level below 1 µg/L through an analysis of microalga toxicity, the quality should be guaranteed [37]. In addition, Choi et al. [40] detected the presence of some bacteria isolated from *A. platensis*, including *Leucobacter* sp., *Aeromicrobium* sp., *Staphylococcus* spp. and *Halomonas* spp. The first four genera were suggested to come from human skin, and thus, their contamination would occur during the subculturing process, whereas contamination with *Halomonas* spp. would possibly result from collected water samples. Thus, controlling and treating the cultivation medium reduce the presence of such contaminants in microalga biomass, preventing them from contaminating broiler meat. Regarding consumer acceptability, the impact of Spirulina on meat quality attributes such as flavour, colour and texture is significant. Research by Park et al. [8] suggests that moderate Spirulina levels can enhance meat colour, attributed to the increased carotenoid content in Spirulina. However, higher levels of Spirulina might lead to changes in flavour or texture that might not be universally preferred [9], underscoring the importance of balancing nutritional enhancements with sensory qualities for consumer acceptance. Furthermore, the perception of Spirulina as a natural and healthful feed additive, as indicated in the studies by Bonos et al. [3], can positively influence consumer preferences, especially in markets favouring natural or functional foods.

The long-term safety and acceptability of high Spirulina inclusion levels, however, remain less clear, because studies including high levels (equal to or above 10%) of Spirulina are scarce. Short-term studies, such as those conducted by Pestana et al. [9], indicate beneficial effects, with some negative effects on growth performance, but the long-term consumption of meat from broilers fed high levels of Spirulina over extended periods requires further investigation. Continuous research and monitoring are essential to ensuring no adverse effects on meat quality or consumer health, as well as understanding the cumulative effects of compounds like carotenoids and fatty acids on human health.

Highlighting Spirulina’s nutritional composition (protein, vitamins and antioxidants) and ensuring and advancing towards sustainable microalga production, as well as providing clear and informative labelling with the positive aspects of Spirulina for consumers, which would allow for an evaluation of their willingness to pay a higher price for premium food, are important aspects for stakeholders in poultry industry [41]. 

In conclusion, while the inclusion of Spirulina in broiler diets presents potential health benefits, the broader implications, including safety and consumer acceptability, must be carefully considered. Ongoing research, including a thorough understanding of Spirulina’s long-term effects, is essential to ensuring that Spirulina-enhanced broiler meat remains a safe and acceptable option for consumers, particularly at higher inclusion levels and over extended feeding durations.

**Figure 1:**
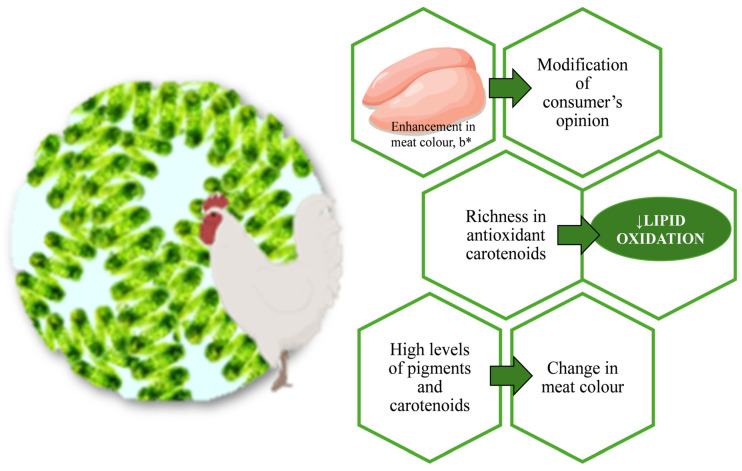
Effect of cumulative Spirulina intake on broiler meat quality, nutritional and health-related attributes. Each arrow represents the effect of each aspect; b*: yellowness (CIELAB scale).

Figure 1 summarises the results reviewed in this work.

## 6. Conclusions and Future Research

This study delved into the multifaceted effects of cumulative Spirulina intake on broiler meat traits, uncovering both its significant benefits and intricate complexities. The research highlights Spirulina’s notable contribution to enhancing meat colour and its overall nutritional profile, particularly at moderate intake levels. A critical observation from this study is the saturation effect in colour enhancement, where initial increases in Spirulina intake lead to significant improvements, but these benefits plateau at higher cumulative intake levels. This finding is crucial for understanding the dose-dependent effects of Spirulina.

Furthermore, this study demonstrated Spirulina’s positive impact on the fatty acid profile and antioxidant capacity of broiler meat. Notably, an increase in beneficial *n* − 3 PUFAs and a reduction in lipid oxidation were observed with increased Spirulina intake. However, these effects are not strictly linear, particularly at higher intake levels, suggesting a nuanced interaction between the Spirulina dosage and meat quality.

The complexities are particularly evident when considering the sensory aspects of meat, such as flavour and texture. While moderate cumulative levels of Spirulina intake positively affect these sensory attributes, higher levels might induce changes that are not universally preferred by consumers, such as the yellowness of meat and the presence of off-flavours. The evaluation of sensory aspects can be performed using a trained sensory panel. This finding underscores the importance of identifying optimal cumulative Spirulina intake levels that strike a balance between nutritional enhancement and consumer acceptability.

In terms of safety and regulatory compliance, this study emphasises the necessity of ensuring Spirulina’s purity and adherence to regulatory standards to maintain consumer trust and market access. Future research should prioritise long-term studies to further explore the effects of high Spirulina intake levels over extended periods and explore the optimal Spirulina level for sustainable poultry farming practices with the best cost efficiency. This approach is vital for a comprehensive understanding of Spirulina’s impact on meat quality and its cumulative effects on consumer health. Also, other species of Spirulina with their pros and cons in feeding poultry should be evaluated in relevant research in the near future.

## Data Availability

The original contributions presented in the study are included in the article, further inquiries can be directed to the corresponding author.
